# Long-term risk of adverse outcomes according to atrial fibrillation type

**DOI:** 10.1038/s41598-022-05688-9

**Published:** 2022-02-09

**Authors:** Steffen Blum, Stefanie Aeschbacher, Michael Coslovsky, Pascal B. Meyre, Philipp Reddiess, Peter Ammann, Paul Erne, Giorgio Moschovitis, Marcello Di Valentino, Dipen Shah, Jürg Schläpfer, Rahel Müller, Jürg H. Beer, Richard Kobza, Leo H. Bonati, Elisavet Moutzouri, Nicolas Rodondi, Christine Meyer-Zürn, Michael Kühne, Christian Sticherling, Stefan Osswald, David Conen, the BEAT-AF and Swiss-AF investigators, Stefanie Aeschbacher, Chloé Auberson, Steffen Blum, Leo Bonati, Selinda Ceylan, David Conen, Simone Evers-Doerpfeld, Ceylan Eken, Marc Girod, Elisa Hennings, Elena Herber, Vasco Iten, Philipp Krisai, Maurin Lampart, Mirko Lischer, Christine Meyer-Zürn, Pascal Meyre, Andreas U. Monsch, Christian Müller, Rebecca E. Paladini, Anne Springer, Christian Sticherling, Thomas Szucs, Gian Völlmin, Stefan Osswald, Michael Kühne, Drahomir Aujesky, Urs Fischer, Juerg Fuhrer, Laurent Roten, Simon Jung, Heinrich Mattle, Seraina Netzer, Luise Adam, Carole Elodie Aubert, Martin Feller, Axel Loewe, Elisavet Moutzouri, Claudio Schneider, Tanja Flückiger, Cindy Groen, Lukas Ehrsam, Sven Hellrigl, Alexandra Nuoffer, Damiana Rakovic, Nathalie Schwab, Rylana Wenger, Tu Hanh Zarrabi Saffari, Nicolas Rodondi, Tobias Reichlin, Christopher Beynon, Roger Dillier, Michèle Deubelbeiss, Franz Eberli, Christine Franzini, Isabel Juchli, Claudia Liedtke, Samira Murugiah, Jacqueline Nadler, Thayze Obst, Jasmin Roth, Fiona Schlomowitsch, Xiaoye Schneider, Katrin Studerus, Noreen Tynan, Dominik Weishaupt, Andreas Müller, Simone Fontana, Corinne Friedli, Silke Kuest, Karin Scheuch, Denise Hischier, Nicole Bonetti, Alexandra Grau, Jonas Villinger, Eva Laube, Philipp Baumgartner, Mark Filipovic, Marcel Frick, Giulia Montrasio, Stefanie Leuenberger, Franziska Rutz, Jürg-Hans Beer, Angelo Auricchio, Adriana Anesini, Cristina Camporini, Giulio Conte, Maria Luce Caputo, Francois Regoli, Tiziano Moccetti, Roman Brenner, David Altmann, Michaela Gemperle, Peter Ammann, Mathieu Firmann, Sandrine Foucras, Martine Rime, Daniel Hayoz, Benjamin Berte, Virgina Justi, Frauke Kellner-Weldon, Brigitta Mehmann, Sonja Meier, Myriam Roth, Andrea Ruckli-Kaeppeli, Ian Russi, Kai Schmidt, Mabelle Young, Melanie Zbinden, Richard Kobza, Elia Rigamonti, Carlo Cereda, Alessandro Cianfoni, Maria Luisa De Perna, Jane Frangi-Kultalahti, Patrizia Assunta Mayer Melchiorre, Anica Pin, Tatiana Terrot, Luisa Vicari, Giorgio Moschovitis, Georg Ehret, Hervé Gallet, Elise Guillermet, Francois Lazeyras, Karl-Olof Lovblad, Patrick Perret, Philippe Tavel, Cheryl Teres, Dipen Shah, Nathalie Lauriers, Marie Méan, Sandrine Salzmann, Jürg Schläpfer, Alessandra Pia Porretta, Andrea Grêt, Jan Novak, Sandra Vitelli, Frank-Peter Stephan, Jane Frangi-Kultalahti, Augusto Gallino, Luisa Vicari, Marcello Di Valentino, Helena Aebersold, Fabienne Foster, Matthias Schwenkglenks, Jens Würfel, Anna Altermatt, Michael Amann, Marco Düring, Petra Huber, Esther Ruberte, Tim Sinnecker, Vanessa Zuber, Michael Coslovsky, Pascal Benkert, Gilles Dutilh, Milica Markovic, Pia Neuschwander, Patrick Simon, Ramun Schmid

**Affiliations:** 1 Division of Cardiology, Department of Medicine, University Hospital Basel, Basel, Switzerland; 2 Cardiovascular Research Institute Basel, University Hospital Basel, Basel, Switzerland; 3 Division of Cardiology, Kantonsspital St. Gallen, St. Gallen, Switzerland; 4 Laboratory for Signal Transduction, Department of Biomedicine, University of Basel, Basel, Switzerland; 5 Ente Ospedaliero Cantonale Lugano, Lugano, Switzerland; 6 Ente Ospedaliero Cantonale Bellinzona, Belinzona, Switzerland; 7 University Hospital Geneva, Geneva, Switzerland; 8 University Hospital Lausanne, Lausanne, Switzerland; 9 Kantonspital Baden, Baden, Switzerland; 10 Luzerner Kantonsspital, Luzern, Switzerland; 11 Department of Neurology and Stroke Center, University Hospital Basel, Basel, Switzerland; 12 Institute of Primary Health Care (BIHAM), University of Bern, Bern, Switzerland; 13 University Hospital Bern, Bern, Switzerland; 14 Population Health Research Institute, McMaster University, Barton Street East, Hamilton, ON Canada; 15 Stadtspital Triemli, Zurich, Switzerland; 16 Cardiocentro Lugano, Lugano, Switzerland; 17 Hôpital Cantonal Fribourg, Fribourg, Switzerland; 18 University of Zurich/University Hospital Zurich, Zurich, Switzerland; 19 Medical Image Analysis Center AG, Basel, Switzerland; 20 Clinical Trial Unit Basel, Basel, Switzerland; 21 Schiller AG, Baar, Switzerland

**Keywords:** Cardiology, Atrial fibrillation

## Abstract

Sustained forms of atrial fibrillation (AF) may be associated with a higher risk of adverse outcomes, but few if any long-term studies took into account changes of AF type and co-morbidities over time. We prospectively followed 3843 AF patients and collected information on AF type and co-morbidities during yearly follow-ups. The primary outcome was a composite of stroke or systemic embolism (SE). Secondary outcomes included myocardial infarction, hospitalization for congestive heart failure (CHF), bleeding and all-cause mortality. Multivariable adjusted Cox proportional hazards models with time-varying covariates were used to compare hazard ratios (HR) according to AF type. At baseline 1895 (49%), 1046 (27%) and 902 (24%) patients had paroxysmal, persistent and permanent AF and 3234 (84%) were anticoagulated. After a median (IQR) follow-up of 3.0 (1.9; 4.2) years, the incidence of stroke/SE was 1.0 per 100 patient-years. The incidence of myocardial infarction, CHF, bleeding and all-cause mortality was 0.7, 3.0, 2.9 and 2.7 per 100 patient-years, respectively. The multivariable adjusted (a) HRs (95% confidence interval) for stroke/SE were 1.13 (0.69; 1.85) and 1.27 (0.83; 1.95) for time-updated persistent and permanent AF, respectively. The corresponding aHRs were 1.23 (0.89, 1.69) and 1.45 (1.12; 1.87) for all-cause mortality, 1.34 (1.00; 1.80) and 1.30 (1.01; 1.67) for CHF, 0.91 (0.48; 1.72) and 0.95 (0.56; 1.59) for myocardial infarction, and 0.89 (0.70; 1.14) and 1.00 (0.81; 1.24) for bleeding. In this large prospective cohort of AF patients, time-updated AF type was not associated with incident stroke/SE.

## Introduction

Atrial fibrillation (AF) is classified into paroxysmal, persistent or permanent AF^1^. This classification into different AF types is useful in clinical practice, as more sustained forms of the arrhythmia are less amenable to rhythm control treatment strategies. For example, catheter ablation is less successful among patients with persistent AF compared to paroxysmal AF^2^.

Patients with AF face an increased risk of stroke, congestive heart failure (CHF) and death^3–5^. To what extent the risk of these and other outcome events is influenced by AF type is still debated. A recent meta-analysis found a higher risk of thromboembolism and death in patients with non-paroxysmal AF than in patients with paroxysmal AF during 1 to 2.8 years of follow-up^6^. This raises the possibility that the arrhythmia itself is involved in the disease process and may at least in part explain the increased risk for adverse outcome events. On the other hand, patients with non-paroxysmal AF are usually older and have a higher burden of risk factors and co-morbidities than patients with paroxysmal AF^7,8^, suggesting that co-morbidities and not AF type may be key drivers for the increased risk of adverse outcome events.

AF type^9^ and co-morbidities^10^ change over time in a relevant number of AF patients. However, none of the previous studies took these changes into account in their analyses. In this study we therefore assessed the risk of stroke/systemic embolism (SE) and other adverse outcome events according to AF type, taking into consideration changes of AF type and co-morbidities in models with time-varying covariates.

## Methods

To increase sample size and power, we combined data from two ongoing prospective, observational, multicenter cohort studies with similar inclusion and exclusion criteria.

Between 2010 and 2014 a total of 1553 patients with documented AF were included in the Basel Atrial Fibrillation (BEAT-AF) study across 7 centers in Switzerland^11^. The Swiss Atrial Fibrillation (Swiss-AF) study enrolled 2415 AF patients between 2014 and 2017 across 14 centers in Switzerland^12,13^. In both cohorts, patients were required to have previously documented AF. Main exclusion criteria for both cohorts were the inability to sign informed consent, the presence of exclusively short transient episodes of AF (e.g., secondary after cardiac surgery, thyrotoxicosis or severe sepsis), as well as any acute illness within the last 4 weeks. Patients enrolled in BEAT-AF were not eligible for participation in Swiss-AF.

For the present study, we excluded 77 (1.9%) patients from the combined dataset without any follow-up information and 41 (0.9%) because of missing baseline AF type or co-morbidities. Seven (0.2%) patients with accidental inclusion in both cohorts were only counted once, leaving 3843 (97.0%) patients for this analyses, which used follow-up data up to April 17, 2019. The study protocols of both studies were approved by the local ethics committees (Ethikkommission Nordwest und Zentralschweiz), and informed written consent was obtained from all participants. All methods were performed in accordance with relevant guidelines and regulations.

### Baseline and follow-up assessments

Patients completed detailed questionnaires about personal, medical, nutritional and lifestyle factors in both cohorts. Smoking status was categorized as current smokers or non-current smokers (past or never smokers). Body mass index (BMI) was calculated as weight in kg divided by height in meters squared. AF was classified according to the guidelines of the European Society of Cardiology into the three main categories paroxysmal (self-terminating, usually within 48 h), persistent (episodes lasting longer than seven days or requiring termination by electrical or medical cardioversion) or permanent AF (AF is accepted by the patient and the physician and no further attempts to restore sinus rhythm are performed)^1^. Stroke risk was categorized using the CHA_2_DS_2_-VASc score as recommended by current guidelines^1^. The CHA_2_DS_2_-VASc score ranges from 0 to 9 by giving one point for a history of hypertension, CHF, diabetes, vascular disease, age between 65 and 74 years and female sex, and two points for a history of stroke/TIA or an age ≥ 75 years. Coronary artery disease was defined as either a history of myocardial infarction, percutaneous transluminal coronary angioplasty and/or coronary bypass graft surgery.

After a face-to-face baseline examination in both cohorts, yearly follow-up examinations in BEAT-AF were performed by mail and phone interviews, while in Swiss-AF all patients had yearly clinical follow-up visits. During these yearly follow-ups, we obtained updated information about risk factors, co-morbidities, AF type, and intercurrent adverse outcome events. The current AF type was assessed in both cohorts during each baseline and follow-up visit based on the study related information, as well as all available clinical information and medical reports.

### Outcome events and definitions

The same outcome definitions were used in both cohorts. They are provided in Supplementary Table S1. Each event in each cohort was independently validated by two physicians. In case of discrepancy, a third physician was consulted for a final decision. The primary outcome for this analysis was a composite of stroke or SE. Secondary outcomes were myocardial infarction, hospitalization for CHF, bleeding, major adverse cardiovascular events (MACE) and all-cause mortality.

### Statistical analysis

Baseline characteristics were stratified by AF type at study entry. Categorical variables were presented as numbers (percentages) and compared using χ^2^-tests. The distribution of continuous variables was checked using kurtosis, skewness and visual inspection of the histogram. Continuous variables were presented as mean ± standard deviation or median (interquartile range) and compared using analysis of variance or Kruskal–Wallis tests, as appropriate.

To assess the association of clinical predictors with adverse outcome events, we constructed Cox regression models with time-varying covariates to estimate hazard ratios (HR) and 95% confidence intervals (95% CI) and to adjust for potential confounders. All regression models were adjusted for a pre-defined set of baseline covariates, including age, sex, and heart rate, and time-updated information on smoking (current vs. history/never smoker), BMI, diabetes, coronary artery disease, hypertension, CHF, stroke/transient ischemic attack (TIA), renal failure, oral anticoagulation, antiplatelet therapy, electrical cardioversion and pulmonary vein isolation. We used the most recent update of the AF type before the outcome event or the latest follow-up, as appropriate.

A sensitivity analysis was performed using a binary AF type classification of paroxysmal vs. non-paroxysmal (persistent or permanent) AF as these types can be difficult to be distinguished. An interaction analysis using a multiplicative interaction between cohort and AF type was performed for all outcome events. As information on BMI has not been collected during the first two follow-ups in BEAT-AF, we imputed weight, whenever missing, based on a linear mixed effects model which included age, sex, height and a natural spline for time since recruitment as fixed effects, and patient ID as random intercept. The assumption of proportional hazards was assessed visually by plotting log(-log(survival)) against log(time) for categorical variables. Schoenfeld-residuals were plotted for continuous variables. All analyses were conducted using R version 3.5.1 (R Core Team 2018). A two-sided *p* value < 0.05 was considered to indicate statistical significance.

## Results

Baseline characteristics stratified by baseline AF type are shown in Table 1. Mean (± SD) age of the total population was 71 ± 10 years and 1080 (28%) participants were women. At baseline, 1895 (49%) patients had paroxysmal AF, 1046 (27%) persistent AF and 902 (24%) permanent AF. Patients with persistent or permanent AF were older, less often women, and more often had a history of coronary artery disease, hypertension, CHF, diabetes and renal failure than patients with paroxysmal AF (all *p* < 0.001). Among patients with paroxysmal, persistent and permanent AF, mean (± SD) CHA_2_DS_2_-Vasc Score was 3.0 (± 1.8), 3.1 (± 1.7) and 3.9 (± 1.6) respectively (*p* < 0.001), and 76%, 91% and 94% of patients received oral anticoagulation (*p* < 0.001).

**Table 1:** Baseline characteristics stratified by baseline atrial fibrillation type.

Characteristic	Paroxysmal (n = 1895)	Persistent (n = 1046)	Permanent (n = 902)	*p* value
Age, [years ]	70 ± 11	70 ± 10	76 ± 8	< 0.001
Female sex, No. (%)	606 (32.0)	263 (25.1)	211 (23.4)	< 0.001
Body mass index, [kg/m^2^]	27 ± 5	28 ± 5	28 ± 5	< 0.001
Heart rate, [beats/min]	63 [56; 72]	67 [59; 80]	73 [63; 85]	< 0.001
Systolic blood pressure, [mmHg]	135 ± 18	133 ± 19	132 ± 19	< 0.001
History of coronary heart disease, No. (%)	447 (23.6)	259 (24.8)	326 (36.1)	< 0.001
History of stroke/TIA, No. (%)	333 (17.6)	147 (14.1)	176 (19.5)	0.004
History of hypertension, No. (%)	1219 (64.3)	729 (69.7)	692 (76.7)	< 0.001
History of congestive heart failure, No. (%)	298 (15.7)	292 (27.9)	321 (35.6)	< 0.001
History of diabetes mellitus, No. (%)	252 (13.3)	156 (14.9)	194 (21.5)	< 0.001
History of renal failure, No. (%)	286 (15.1)	188 (18.0)	240 (26.6)	< 0.001
History of hyperthyroidism, No. (%)	57 (3.0)	61 (5.8)	54 (6.0)	< 0.001
Current smoker, No. (%)	153 (8.1)	86 (8.2)	58 (6.4)	0.25
Regular physical activity, No. (%)	997 (52.8)	500 (48.1)	369 (41.0)	< 0.001
History of pulmonary vein isolation, No. (%)	474 (25.0)	277 (26.5)	64 (7.1)	< 0.001
History of electrical cardioversion, No. (%)	341 (18.0)	693 (66.3)	295 (32.7)	< 0.001
CHA_2_DS_2_-VASc score	3.0 ± 1.8	3.1 ± 1.7	3.9 ± 1.6	< 0.001
Oral anticoagulation, No. (%)	1442 (76.1)	947 (90.5)	845 (93.7)	< 0.001
Vitamin K-antagonists	747 (39.4%)	491 (46.9)	637 (70.6)	
Non-vitamin K antagonist oral anticoagulants	695 (36.7)	456 (43.6)	208 (23.1)	
Antiplatelet therapy, No. (%)	453 (23.9)	184 (17.6)	195 (21.6)	< 0.001

Data are means ± SD, medians (IQR) or counts (percentages); *p* values were based on ANOVA, Kruskal–Wallis-Tests or Chi-Square tests as appropriate.

*TIA* transient ischemic attack.

During 11,920 patient-years of follow-up, we observed a total of 1177 (14.6%) changes in the AF type. Any AF progression was observed among 490 (12.8%) patients, any AF regression in 343 (8.9%) patients. The overall incidence of stroke/SE was 1.0 per 100 patient-years of follow-up. It was 0.8, 1.0 and 1.5 per 100 patient-years in patients with paroxysmal, persistent and permanent AF (Table 2). In age and sex adjusted models, the HR (95% CI) for stroke/SE was 1.22 (95% CI 0.76; 1.94, *p* = 0.41) for time-updated persistent and 1.35 (95% CI 0.88; 2.05, *p* = 0.17) for time-updated permanent AF. The multivariable adjusted HRs were 1.13 (95% CI 0.69; 1.85), *p* = 0.64 and 1.27 (95% CI 0.84; 2.00), *p* = 0.28, respectively.

**Table 2:** Risk of stroke/systemic embolism according atrial fibrillation type.

Outcome	No. of events	Incidence per 100py	Hazard ratio (95% CI)			
			Age and sex adjusted	*p* value	Multivariable adjusted	*p* value
**Stroke/systemic embolism**						
Paroxysmal	52	0.8	Ref		Ref	
Persistent	27	1.0	1.22 (0.76; 1.94)	0.41	1.13 (0.69; 1.85)	0.64
Permanent	42	1.5	1.35 (0.88; 2.05)	0.17	1.27 (0.83; 1.95)	0.28

Data are hazard ratios (HR) (95% confidence intervals [CI]). p-values were based on Cox regression models.

*No.* number, *Ref.* reference, *py* patient years.

P-values are based on Cox regression models. Multivariable models were adjusted for age, sex, heart rate and time-updated: smoking status (current vs. history/never smoker), BMI, history of diabetes, history of coronary artery disease, history of hypertension, history of heart failure, history of stroke and/or transient ischemic attack, history of renal failure, oral anticoagulation, antiplatelet therapy, history of pulmonary vein isolation and history of electrical cardioversion.

Table 3 shows absolute and relative risks for secondary outcome events according to AF type. The incidence per 100 patients-years was higher among patients with time-updated persistent and permanent AF for all outcome events. In age and sex adjusted models, presence of time-updated persistent AF was significantly associated with CHF hospitalizations (HR 1.47 [95% CI 1.12; 1.93], *p* < 0.001). Presence of time-updated permanent AF was significantly associated with a higher risk of CHF hospitalization (HR 1.78 [95% CI 1.39; 2.26], *p* < 0.001), MACE (HR 1.75 [95% CI 1.37; 2.23], *p* < 0.001), all-cause mortality (HR 1.80 [95% CI 1.40; 2.30], *p* < 0.001), clinically relevant non-major bleeding (HR 1.38 [95% CI 1.07; 1.77], *p* = 0.01) and any bleeding (HR 1.28 [95% CI 1.05; 1.57], *p* = 0.02). The associations were attenuated in multivariable models that adjusted for time-updated co-morbidities, but time-updated persistent AF remained associated with CHF hospitalizations (*p* = 0.05) and presence of time-updated permanent AF remained significantly associated with CHF hospitalization (*p* = 0.04), MACE (*p* = 0.008) and all-cause mortality (*p* = 0.005) (Table 3). All AF type by cohort p-values for interaction in the multivariable models were > 0.05 (data not shown).

**Table 3:** Risk of secondary endpoints according to atrial fibrillation type.

Outcome	No. of events	Incidence, per 100py	Hazard ratio (95% CI)			
			Age and sex adjusted	*p* value	Multivariable adjusted	*p* value
**Congestive heart failure hospitalization**						
Paroxysmal	133	2.1	Ref		Ref	
Persistent	86	3.1	1.47 (1.12; 1.93)	0.006	1.34 (1.00; 1.80)	0.05
Permanent	144	5.5	1.78 (1.39; 2.26)	< 0.001	1.30 (1.01; 1.67)	0.04
**Myocardial infarction**						
Paroxysmal	41	0.6	Ref		Ref	
Persistent	15	0.5	0.81 (0.45; 1.47)	0.49	0.91 (0.48; 1.72)	0.77
Permanent	28	1.0	1.10 (0.67; 1.80)	0.71	0.95 (0.56; 1.59)	0.84
**MACE**						
Paroxysmal	134	2.1	Ref		Ref	
Persistent	73	2.6	1.23 (0.92; 1.64)	0.15	1.15 (0.85; 1.57)	0.37
Permanent	145	5.3	1.75 (1.37; 2.23)	< 0.001	1.41 (1.10; 1.82)	0.008
**All-cause mortality**						
Paroxysmal	122	1.9	Ref		Ref	
Persistent	68	2.4	1.28 (0.95; 1.72)	0.11	1.23 (0.89; 1.69)	0.21
Permanent	147	5.2	1.80 (1.40; 2.30)	< 0.001	1.45 (1.12; 1.87)	0.005
**Major bleeding**						
Paroxysmal	99	1.6	Ref		Ref	
Persistent	51	1.8	1.19 (0.85; 1.68)	0.31	1.11 (0.76; 1.61)	0.59
Permanent	71	2.6	1.17 (0.86; 1.61)	0.32	0.98 (0.71; 1.36)	0.90
**Non-major bleeding**						
Paroxysmal	150	2.4	Ref		Ref	
Persistent	69	2.5	1.03 (0.77; 1.37)	0.86	0.82 (0.60; 1.12)	0.21
Permanent	117	4.5	1.38 (1.07; 1.77)	0.01	1.02 (0.79; 1.33)	0.86
**Any bleeding**						
Paroxysmal	237	3.9	Ref		Ref	
Persistent	112	4.1	1.06 (0.85; 1.33)	0.61	0.89 (0.70; 1.14)	0.37
Permanent	174	6.9	1.28 (1.05; 1.57)	0.02	1.00 (0.81; 1.24)	1.00

Data are hazard ratios (HR) (95% confidence intervals [CI]). p-values were based on Cox regression models.

*No.* number, *Ref.* reference, *py* patient years, *MACE* major adverse cardiovascular event (Ischaemic stroke/myocardial infarction/cardiovascular death). P-values are based on Cox regression models. Multivariable models were adjusted for age, sex, heart rate and time-updated: smoking status (current vs. history/never smoker), BMI, history of diabetes, history of coronary artery disease, history of hypertension, history of heart failure, history of stroke and/or transient ischemic attack, history of renal failure, oral anticoagulation, antiplatelet therapy, history of pulmonary vein isolation and history of electrical cardioversion.

Independent predictors for the primary outcome stroke/SE were history of stroke/TIA (HR 2.70 [95% CI 1.88; 3.89], *p* < 0.001), increasing age (HR 1.05 per year [95% CI 1.03; 1.08], *p* < 0.001), history of CHF (HR 1.51 [95% CI 1.01; 2.24], *p* = 0.04) and oral anticoagulation (HR 0.59 [95% CI 0.35; 1.00], *p* = 0.05). HRs for all covariates of primary and secondary outcomes are presented in the Supplementary Table S2.

The results remained broadly consistent when persistent and permanent AF were combined in time-updated non-paroxysmal AF for a binary sensitivity analysis (Supplementary Table S3).

## Discussion

In this large study of well-treated patients with AF, the incidence rate of stroke/SE among patients with time-updated permanent AF was almost twice as high compared with patients with paroxysmal AF. Adjustment for age and sex weakened this association and after taking into account the effect of risk factors, co-morbidities and their changes over time, the association of AF type and stroke/SE was further attenuated. Major confounding was also observed for all secondary outcome events, although time-updated AF type remained significant association with some of these outcomes. These results suggest that co-morbidities have a significant impact on the relationships between AF type and risk of adverse outcomes.

With an incidence of 1.0 cases per 100 patient-years the risk of stroke/SE was low and overall comparable to stroke rates in well-treated patients from contemporary clinical trials^14–17^. Overall, stroke rates across different cohort studies are highly heterogeneous^18^. The low incidence in our study can be explained at least in part by the high prevalence of oral anticoagulation. At baseline more than 84% of the whole study population and more than 90% of patients classified as having non-paroxysmal AF received oral anticoagulation, and this prevalence tends to go up over time^19^.

In adjusted models with time-varying covariates, time-updated persistent and permanent AF were not significantly associated with stroke/SE. A recent meta-analysis found a pooled adjusted HR for thromboembolism in patients with non-paroxysmal AF of 1.38 (95% CI 1.91; 1.61), *p* < 0.001^6^. It is important to note that none of the studies that has been included in the meta-analysis updated AF type nor co-morbidities. Especially not updating co-morbidities and risk-factor might exacerbate the problem of residual confounding and may lead to an overestimation of the effect of AF type, given the frequent changes in patients with AF^10^. Also, the effect was strongest among patients without oral anticoagulation^6^. Taken together, our results suggest that the independent effect of AF type on stroke risk is modest at best.

On the other hand, several risk factors of the CHA_2_DS_2_-VASc Score were independently associated with stroke/SE in our study (Table S2). Yoon et al. assessed dynamic changes of the CHA_2_DS_2_-VASc Score and the risk of ischemic stroke in a nationwide cohort of more than 160,000 AF patients. They found an annual increase of the CHA_2_DS_2_-VASc Score of 0.14 points and rates of stroke increased when patients accumulated risk factors^10^. Another study on changes in risk factors over time among AF patients showed that 52% of the whole cohort and 90% of patients who experienced a stroke during follow-up developed at least one new CHA_2_DS_2_-VASc risk factor. The most common new-onset risk factor was hypertension, followed by CHF^20^. Therefore clinicians should be aware of the clinical importance to account for dynamic changes of the co-morbidities when assessing the stroke risk in AF patients. Taken together, while presence of persistent and permanent AF might be a mirror of a higher risk factor burden, it should not be used to guide anticoagulation treatment in AF patients.

In multivariable models, presence of time-updated permanent AF was significantly associated all-cause mortality, hospitalization for CHF and MACE. The association with mortality could be driven by more severe strokes, which have been described among patients with persistent and permanent AF^21,22^, but also through new-onset or worsening CHF^4^. In addition, cardiovascular risk factors and co-morbidities such as renal failure, diabetes, history of CHF and age were more prevalent in non-paroxysmal AF patients (Table 1) and also strongly associated with all outcome events (Table S2). Therefore, optimal risk factor control and optimal treatment of co-morbidities remains a major target to prevent adverse outcome events among AF patients.

The absolute risks of CHF hospitalizations, all-cause mortality and bleeding were much higher than the risk of stroke/SE. While better risk prediction and effective treatment with oral anticoagulation led to a significant decline of stroke risk over the last decades^23^, the improvement in mortality was only modest^23,24^ and the outcomes of CHF have not improved at all^25^. It is well known that AF is an independent risk factor for all-cause, cardiovascular and non-cardiovascular death^5^ and the majority of deaths among anticoagulated AF patients were related to CHF^26^. Previous data showed that modifiable risk factors, including obesity, smoking, hypertension and diabetes accounted for the majority of CHF risk in AF patients^27^. Importantly, once AF patients developed CHF, the risk of death significantly increased^27^. Time-updated persistent and permanent AF were independently associated with hospitalization for CHF and time-updated permanent AF was additionally associated with all-cause mortality and MACE suggesting that the AF type might be helpful in risk assessment of these secondary outcome events. In order to address the excess in mortality and morbidity beyond thromboembolism a more holistic and integrated approach to AF management has been advocated^28,29^ and been incorporated into the current guidelines for AF management^30^, aiming to not only avoid stroke, but also improve management of symptoms as well as cardiovascular risk factors and co-morbidities. Adherence to this pathway has shown to significantly reduce major adverse outcome events, especially among frail patients^31^.

One of the key strengths of this study is the large sample size, the fact that all outcome events were independently validated by two physicians, and that co-morbidities and AF type were updated on a yearly basis. On the other hand, some potential limitations need to be taken into account in the interpretation of our results. Due to the observational study design, we are not able to prove causality and residual confounding may persist despite comprehensive multivariable adjustment. Sex-specific analyses were not performed given the relatively low number of women enrolled in our cohorts. The assessment of the clinical AF type is challenging, and does not well correlate with AF burden^32^. However, assessment of AF type is very commonly performed in clinical practice, and our results therefore are of clinical interest.

## Conclusion

In this large, prospective study of contemporary AF patients, presence of time-updated persistent or permanent AF was not associated with an increased risk of stroke/SE in models that accounted for changes in AF type and co-morbidities. While time-updated AF type remained significantly associated with all-cause mortality and CHF hospitalizations, taking into account co-morbidities and risk factors attenuated these results. Therefore, better risk-factor control and management of co-morbidities might help to reduce the incidence of cardiovascular outcomes and mortality among AF patients.

## Supplementary Information

Supplementary Information.

## Data Availability

The patient informed consent forms, as approved by the responsible ethics committee (Ethikkommission Nordwest und Zentralschweiz), do not allow the data to be made publicly available. The participants signed a consent form, which states that their data, containing personal and medical information, are exclusively available for research institutions in an anonymized form. Researchers interested in obtaining the data for research purposes can contact the Swiss-AF scientific lead. Contact information is provided on the Swiss-AF website (http://www.swissaf.ch/contact.htm). Authorization of the responsible ethics committee is mandatory before the requested data can be transferred to external research institutions.
