# Supplementary material for: Long-term risk of adverse outcomes according to atrial fibrillation type
Source: Sci Rep. 2022 Feb 9;12:2208. doi: 10.1038/s41598-022-05688-9 (PMC8828824; doi:10.1038/s41598-022-05688-9)
Supplement: Supplementary file 1 — Supplementary Information. [file 41598_2022_5688_MOESM1_ESM.pdf]

Supplementary Appendix

Long-term risk of adverse outcomes according to atrial fibrillation type

Steffen Blum, Stefanie Aeschbacher, Michael Coslovsky, Pascal Meyre, Philipp Reddiess, Peter Ammann, Paul Erne, Giorgio Moschovitis, Marcello Di Valentino, Dipen Shah, Jürg Schläpfer, Rahel Müller, Jürg H. Beer, Richard Kobza, Leo H. Bonati, Elisavet Moutzouri, Nicolas Rodondi, Christine Meyer-Zürn, Michael Kühne, Christian Sticherling, Stefan Osswald, David Conen

Supplementary Table S1: Definition of outcome events

Stroke	Stroke was further subdivided into ischemic stroke, intracerebral hemorrhage or undetermined strokes. Ischemic stroke was defined as an acute focal neurologic deficit of vascular origin, confirmed by imaging or pathological evidence. Intracerebral hemorrhage was defined as a rapid onset of focal or global neurological dysfunction and/or headache attributable to a focal collection of blood within the brain parenchyma or ventricular system that is not caused by trauma. If strokes fulfilled the above criteria but could not be assigned to an ischemic or hemorrhagic stroke, those events were classified as undetermined stroke. SE was considered as an abrupt occlusion of a systemic artery with objective evidence supporting the diagnosis
Death	Deaths were classified as cardiovascular or non-cardiovascular origin. All deaths were assumed to be of cardiovascular origin unless a non-cardiovascular cause could be clearly established.
Myocardial Infarction	Myocardial infarction was defined according to the universal definition of MI[1] as rise and/or fall of cardiac troponin with at least one value above the 99th percentile of the upper reference limit in a clinical setting consistent with myocardial ischemia, and with at least one of the following:<br>- Symptoms of ischemia<br>- New ST elevation at the J point in two contiguous leads >0.1 mV except for V2-V3. For leads V2-V3 the following cut points apply: ≥0.2 mV in men ≥40 years, ≥0.25 mV in men <40 years and ≥0.15 mV in women<br>- New horizontal or down-sloping ST depression ≥0.05 mV in two contiguous leads and/or T inversion ≥0.1 mV in two contiguous leads with prominent R wave or R/S ratio >1<br>- new left bundle brunch block on ECG<br>- Development of pathological Q waves in the ECG<br>- Imaging evidence of new loss of viable myocardium or new regional wall motion abnormality<br>- Identification of an intracoronary thrombus by angiography or autopsy
Bleeding	Bleedings were classified as major bleedings[2] or clinically relevant non-major bleedings[3] according to the ISTH criteria.<br>Major bleeding was defined as 1.) fatal bleeding or 2.) a symptomatic bleeding in a critical organ or 3.) a bleeding leading to a reduction in hemoglobin ≥ 20 g/L (2.0 g/dL) within the first 7 days after hospital admission or requiring transfusion of at least 2 units of blood or packed red blood cells

	Clinically relevant non-major bleeding was defined as a bleeding event that has been clinically overt, that satisfies none of the additional criteria required for the event to be adjudicated as a major bleeding event, that leads to either:<br>1) hospital admission for bleeding or<br>2) physician guided medical or surgical treatment for bleeding or<br>3) a change in antithrombotic therapy.
Hospitalization for congestive heart failure	Hospitalization for CHF was defined as any hospitalization for acute HF that was associated with at least one overnight stay
Major adverse cardiovascular event	Major adverse cardiovascular event was defined as a combined endpoint consisting of either ischemic stroke, myocardial infarction or cardiovascular death.
Systemic embolism	A systemic arterial embolism was considered to have occurred where there was a clear evidence of abrupt occlusion of a systemic artery consistent with an embolic event.<br><br>Requirements were:<br><br>1. Clinical signs and symptoms consistent with embolic arterial occlusion<br>2. At least one of the following objective findings:<br>- Surgical report indicating evidence of arterial embolism<br>- Pathological specimens related to embolism removal<br>- Imaging evidence consistent with arterial embolism<br>- Autopsy reports

Supplementary Table S2: Full table of predictors and various outcomes

Predictor	Stroke/SE		Congestive heart failure hospitalization		Myocardial Infarction		MACE		All cause death	
	HR (95% CI)	p-value	HR (95% CI)	p-value	HR (95% CI)	p-value	HR (95% CI)	p-value	HR (95% CI)	p-value
Paroxysmal AF	Ref.		Ref.		Ref.		Ref.		Ref.	
Persistent AF	1.13 (0.69; 1.85)	0.64	1.34 (1.00; 1.80)	0.05	0.91 (0.48; 1.72)	0.77	1.15 (0.85; 1.57)	0.37	1.23 (0.89; 1.69)	0.21
Permanent AF	1.27 (0.83; 1.95)	0.28	1.30 (1.01; 1.67)	0.04	0.95 (0.56; 1.59)	0.84	1.41 (1.10; 1.82)	0.008	1.45 (1.12; 1.87)	0.005
Age	1.05 (1.03; 1.08)	<0.001	1.04 (1.03; 1.06)	<0.001	1.05 (1.02; 1.08)	0.004	1.05 (1.03; 1.06)	<0.001	1.05 (1.04; 1.07)	<0.001
Female sex	1.08 (0.73; 1.59)	0.70	1.02 (0.80; 1.29)	0.87	0.98 (0.59; 1.62)	0.93	0.85 (0.66; 1.09)	0.21	0.68 (0.52; 0.88)	0.004
Current smoking	1.34 (0.73; 2.47)	0.34	1.27 (0.86; 1.86)	0.23	1.00 (0.43; 2.35)	1.00	1.12 (0.75; 1.67)	0.59	1.51 (1.03; 2.22)	0.03
History of heart failure	1.51 (1.02; 2.24)	0.04	2.32 (1.86; 2.90)	<0.001	1.03 (0.63; 1.67)	0.92	2.20 (1.75; 2.76)	<0.001	2.60 (2.06; 3.28)	<0.001
Heart rate	1.00 (0.99; 1.01)	0.48	1.01 (1.00; 1.01)	0.008	1.00 (0.98; 1.01)	0.77	1.01 (1.00; 1.01)	0.09	1.01 (1.00; 1.01)	0.005
BMI	0.98 (0.94; 1.02)	0.38	1.02 (1.00; 1.04)	0.06	1.06 (1.01; 1.11)	0.01	1.00 (0.97; 1.02)	0.84	0.99 (0.96; 1.01)	0.35
History of CAD	1.03 (0.67; 1.57)	0.90	1.20 (0.94; 1.53)	0.14	2.48 (1.52; 4.06)	<0.001	1.25 (0.98; 1.59)	0.07	1.01 (0.79; 1.29)	0.93
History of stroke/TIA	2.70 (1.88; 3.89)	<0.001	1.10 (0.85; 1.42)	0.48	1.28 (0.76; 2.14)	0.36	1.41 (1.10; 1.82)	0.007	1.06 (0.81; 1.38)	0.69
History of hypertension	1.04 (0.68; 1.59)	0.87	0.97 (0.73; 1.28)	0.81	1.29 (0.69; 2.41)	0.42	1.11 (0.84; 1.47)	0.46	0.98 (0.74; 1.31)	0.91
History of diabetes	1.47 (0.95; 2.28)	0.08	1.67 (1.32; 2.12)	<0.001	1.81 (1.12; 2.93)	0.02	1.73 (1.35; 2.20)	<0.001	1.93 (1.52; 2.45)	<0.001
History of ECV	1.12 (0.75; 1.68)	0.57	0.83 (0.65; 1.06)	0.13	0.90 (0.54; 1.51)	0.69	0.95 (0.74; 1.22)	0.69	0.90 (0.70; 1.17)	0.44
Oral anticoagulation	0.59 (0.35; 1.00)	0.05	1.29 (0.85; 1.94)	0.23	0.58 (0.30; 1.13)	0.11	0.76 (0.54; 1.08)	0.12	0.58 (0.41; 0.82)	0.002
History of renal failure	0.96 (0.62; 1.47)	0.84	2.33 (1.86; 2.92)	<0.001	1.32 (0.81; 2.15)	0.27	1.47 (1.16; 1.86)	0.001	2.04 (1.61; 2.58)	<0.001
History PVI	1.00 (0.61; 1.64)	0.99	0.52 (0.35; 0.75)	<0.001	0.31 (0.13; 0.76)	0.01	0.69 (0.49; 0.98)	0.04	0.48 (0.32; 0.73)	<0.001
Antiplatelet medication	1.00 (0.61; 1.64)	0.99	0.87 (0.64, 1.17)	0.36	0.87 (0.49; 1.54)	0.62	1.08 (0.80; 1.44)	0.62	0.86 (0.62: 1.17)	0.33

Data are hazard ratios (HR) (95% confidence intervals [CI]). p-values were based on Cox regression models. ECV=electrical cardioversion; No.=number; Ref.=reference; py=patient years. Multivariable models were adjusted for age, sex, heart rate and (*time updated): smoking status (current vs. history/never smoker), BMI, history of diabetes, history of coronary artery disease, history of hypertension, history of heart failure, history of stroke and/or transient ischemic attack, history of renal failure, oral anticoagulation, antiplatelet therapy, history of pulmonary vein isolation and history of electrical cardioversion

Supplementary Table S2 continued

Predictor	Major bleeding		Non-major bleeding		Any bleeding	
	HR (95% CI)	p-value	HR (95% CI)	p-value	HR (95% CI)	p-value
Paroxysmal AF	Ref.		Ref.		Ref.	
Persistent AF	1.11 (0.76; 1.61)	0.59	0.82 (0.60; 1.12)	0.21	0.89 (0.70; 1.14)	0.37
Permanent AF	0.98 (0.71; 1.36)	0.90	1.02 (0.79; 1.33)	0.86	1.00 (0.81; 1.24)	1.00
Age	1.04 (1.02; 1.06)	<0.001	1.04 (1.03; 1.06)	<0.001	1.04 (1.03; 1.06)	<0.001
Female sex	1.06 (0.79; 1.42)	0.70	0.69 (0.54; 0.90)	0.005	0.84 (0.69; 1.03)	0.09
Current smoking	1.29 (0.80; 2.09)	0.30	1.47 (1.01; 2.14)	0.04	1.37 (1.01; 1.86)	0.05
History of heart failure	1.33 (0.99; 1.79)	0.06	1.34 (1.05; 1.70)	0.02	1.31 (1.08; 1.59)	0.006
Heart rate	1.00 (1.00; 1.01)	0.28	1.01 (1.00; 1.01)	0.01	1.01 (1.00; 1.01)	0.01
BMI	0.97 (0.94; 1.00)	0.05	1.01 (0.99; 1.04)	0.24	1.00 (0.98; 1.02)	0.94
History of CAD	1.10 (0.80; 1.52)	0.55	0.85 (0.65; 1.12)	0.25	0.98 (0.79; 1.21)	0.87
History of stroke/TIA	1.25 (0.91; 1.72)	0.17	1.15 (0.89; 1.50)	0.29	1.21 (0.98; 1.49)	0.07
History of hypertension	1.28 (0.90; 1.81)	0.17	1.06 (0.81; 1.39)	0.68	1.19 (0.95; 1.48)	0.13
History of diabetes	1.27 (0.91; 1.78)	0.16	0.87 (0.65; 1.17)	0.36	1.01 (0.80; 1.27)	0.95
History of cardioversion	0.98 (0.71; 1.34)	0.88	1.12 (0.87; 1.43)	0.38	1.08 (0.89; 1.32)	0.45
Oral anticoagulation	1.49 (0.90; 2.47)	0.12	3.63 (2.21; 5.97)	<0.001	2.36 (1.65; 3.37)	<0.001
History of renal failure	1.46 (1.07; 1.99)	0.02	1.12 (0.86; 1.46)	0.39	1.16 (0.94; 1.44)	0.16
History PVI	0.58 (0.38; 0.90)	0.01	0.77 (0.56; 1.06)	0.11	0.73 (0.56; 0.94)	0.02
Antiplatelet medication	1.05 (0.70; 1.57)	0.82	1.53 (1.11; 2.10)	0.009	1.29 (1.00; 1.66)	0.05

Data are hazard ratios (HR) (95% confidence intervals [CI]). p-values were based on Cox regression models. No.=number; Ref.=reference; py=patient years. Multivariable models were adjusted for age, sex, heart rate and time-updated: smoking status (current vs. history/never smoker), BMI, history of diabetes, history of coronary artery disease, history of hypertension, history of heart failure, history of stroke and/or transient ischemic attack, history of renal failure, oral anticoagulation, antiplatelet therapy, history of pulmonary vein isolation and history of electrical cardioversion

Supplemental Table S3: Risk of outcome events according to paroxysmal vs. non-paroxysmal atrial fibrillation

Outcome	No. of events	Incidence	HR (95% CI) age and sex adjusted	p-value	HR (95% CI) multivariable adjusted	p-value
**Stroke/SE**						
paroxysmal	52	0.8/100py	Ref.		Ref.	
non-paroxysmal	69	1.2/100py	1.29 [0.90; 1.86]	0.17	1.27 [0.85; 1.89]	0.25
**Congestive heart failure hospitalization**						
paroxysmal	133	2.1/100py	Ref.		Ref.	
non-paroxysmal	230	4.3/100py	1.64 (1.32; 2.04)	<0.001	1.31 (1.04; 1.65)	0.02
**Myocardial infarction**						
paroxysmal	41	0.6/100py	Ref.		Ref.	
non-paroxysmal	43	0.8/100py	0.97 (0.63; 1.50)	0.90	0.93 (0.58; 1.49)	0.78
**MACE**						
paroxysmal	134	2.1/100py	Ref.		Ref.	
non-paroxysmal	218	3.9/100py	1.52 (1.22; 1.89)	<0.001	1.32 (1.04; 1.67)	0.02
**All-cause mortality**						
paroxysmal	122	1.9/100py	Ref.		Ref.	
non-paroxysmal	215	3.8/100py	1.58 (1.26; 1.98)	<0.001	1.38 (1.08; 1.75)	0.009
**Major bleeding**						
paroxysmal	99	1.6/100py	Ref.		Ref.	
non-paroxysmal	122	2.2/100py	1.18 (0.90; 1.55)	0.22	1.03 (0.77; 1.37)	0.86
**Non-major bleeding**						
paroxysmal	150	2.4/100py	Ref.		Ref.	
non-paroxysmal	186	3.4/100py	1.21 (0.98; 1.51)	0.08	0.94 (0.74; 1.19)	0.61
**Any bleeding**						
paroxysmal	237	3.9/100py	Ref.		Ref.	
non-paroxysmal	286	5.4/100py	1.18 (0.99; 1.40)	0.06	0.96 (0.79; 1.16)	0.66

Data are hazard ratios (HR) (95% confidence intervals [CI]). p-values were based on Cox regression models. SE=systemic embolism; No.=number; Ref.=reference. py=patient years. MACE=major adverse cardiovascular event (Ischaemic stroke/myocardial infarction/cardiovascular death). Multivariable models were adjusted for age, sex, heart rate and time updated: smoking status (current vs. history/never smoker), BMI, history of diabetes, history of coronary artery disease, history of hypertension, history of heart failure, history of stroke and/or transient ischemic attack, history of renal failure, oral anticoagulation, antiplatelet therapy, history of pulmonary vein isolation and history of electrical cardioversion

Supplemental References

1 Thygesen, K. *et al.* Third universal definition of myocardial infarction. *J Am Coll Cardiol* **60**, 1581-1598, doi:10.1016/j.jacc.2012.08.001 (2012).

2 Schulman, S. & Kearon, C. Definition of major bleeding in clinical investigations of antihemostatic medicinal products in non-surgical patients. *J Thrombos Haemost* **3**, 692-694, doi:10.1111/j.1538-7836.2005.01204.x (2005).

3 Kaatz, S., Ahmad, D., Spyropoulos, A. C. & Schulman, S. Definition of clinically relevant non-major bleeding in studies of anticoagulants in atrial fibrillation and venous thromboembolic disease in non-surgical patients: communication from the SSC of the ISTH. *J Thromb Haemost* **13**, 2119-2126 (2015).
